# Antimicrobial Resistance: Two-Component Regulatory Systems and Multidrug Efflux Pumps

**DOI:** 10.3390/antibiotics12060965

**Published:** 2023-05-26

**Authors:** Giuseppe Valerio De Gaetano, Germana Lentini, Agata Famà, Francesco Coppolino, Concetta Beninati

**Affiliations:** 1 Department of Human Pathology, University of Messina, 98124 Messina, Italy; 2 Department of Biomedical, Dental and Imaging Sciences, University of Messina, 98124 Messina, Italy; 3 Scylla Biotech Srl, 98124 Messina, Italy

**Keywords:** antibiotic resistance, multidrug efflux pumps, TCS, transcriptional regulation

## Abstract

The number of multidrug-resistant bacteria is rapidly spreading worldwide. Among the various mechanisms determining resistance to antimicrobial agents, multidrug efflux pumps play a noteworthy role because they export extraneous and noxious substrates from the inside to the outside environment of the bacterial cell contributing to multidrug resistance (MDR) and, consequently, to the failure of anti-infective therapies. The expression of multidrug efflux pumps can be under the control of transcriptional regulators and two-component systems (TCS). TCS are a major mechanism by which microorganisms sense and reply to external and/or intramembrane stimuli by coordinating the expression of genes involved not only in pathogenic pathways but also in antibiotic resistance. In this review, we describe the influence of TCS on multidrug efflux pump expression and activity in some Gram-negative and Gram-positive bacteria. Taking into account the strict correlation between TCS and multidrug efflux pumps, the development of drugs targeting TCS, alone or together with already discovered efflux pump inhibitors, may represent a beneficial strategy to contribute to the fight against growing antibiotic resistance.

## 1. Introduction

**Figure 1:**
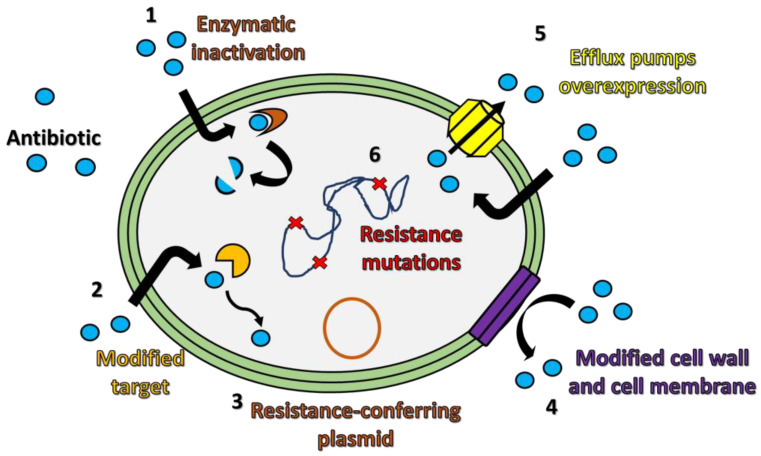
Mechanisms of antibiotic resistance. A simplified bacterium is depicted in which various mechanisms for resistance to antimicrobial substances are highlighted. A generic antibiotic is represented as a blue ball and its fate, after having contact with the bacterium, is analyzed in six potential occasions: (1) drug inactivation by enzymes; (2) drug target modification; (3) plasmid-carrying genes conferring antibiotic resistance; (4) changes in drug permeability following cell wall and cell membrane modifications; (5) expression of efflux pumps that expel drugs outside the bacterial cell; (6) selective DNA mutations altering the bacterial genome expression.

Multidrug resistance (MDR) is increasingly becoming a serious problem due to the capacity of numerous bacteria to develop mechanisms of defense against antimicrobial chemotherapy [1,2,3]. From a biochemical point of view, resistance to antibiotics often consists of the inability of an antibiotic to reach the microbial targets at a satisfactory concentration to completely inhibit their biological functions. Bacteria showing resistance to single or multiple drugs are considered as “superbugs” [4,5]. Causes related to this growing phenomenon are usually associated with the ability of pathogenic bacteria to transfer genes conferring drug resistance among different bacterial species, but also with the inappropriate prescription of antimicrobial therapies in medical treatments, which provides resistant bacteria with a selective advantage [6,7]. Mechanisms of bacterial antibiotic resistance can involve (i) inactivation of the antibiotic by enzymatic modification, (ii) reduction in antibiotic affinity for its molecular bacterial targets, (iii) transfer of plasmids carrying MDR-related genes from one species to another; (iv) alterations in the permeability of the bacterial cell surface, (v) overexpression of active efflux pumps recognizing various antibiotics with different mechanisms of action, and (vi) the presence of polymorphisms or insertions in the bacterial genome at the level of DNA sequence encoding for transcriptional regulators (Figure 1) [8,9].

**Figure 2:**
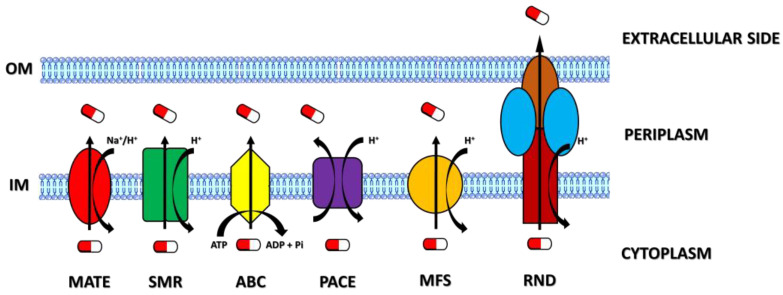
Schematic representations of the main families of multidrug efflux pumps. Each transport system is depicted with a different shape and color. The source of energy needed for substrate transport is further indicated. A generic antibiotic is represented as a pill. The transporters indicated in the figure are the multidrug and toxic compound extrusion (MATE) family, the small multidrug resistance (SMR) family, the ATP-binding cassette (ABC) family, the proteobacterial antimicrobial compound efflux (PACE) family, the major facilitator superfamily (MFS), and the resistance-nodulation-division (RND) family. The principal sites where efflux pumps may be found located in Gram-negative or Gram-positive bacteria are the outer membrane or the inner membrane that, in the figure, are indicated with the acronyms OM and IM, respectively.

Efflux pumps are transporters playing important roles in bacterial pathogenesis, metabolism, and multidrug resistance by decreasing the concentrations of intracellular extraneous substances (i.e., antibiotics, disinfectants, detergents, etc.) and preventing them from reaching their biological targets [10,11,12,13]. For this reason, efflux transporters represent attractive targets for the development of new inhibitors that can help clinicians fight against MDR-linked infectious diseases. On the basis of their sequence similarity, substrate affinity, structure, and energy source, efflux transporters are mainly grouped into five superfamilies: the multidrug and toxic compound extrusion (MATE) superfamily, small multidrug resistance (SMR) superfamily, ATP-binding cassette (ABC) superfamily, proteobacterial antimicrobial compound efflux (PACE) superfamily, major facilitator superfamily (MFS), and resistance nodulation and cell division (RND) superfamily [14,15]. While transporters of the MATE, MFS, RND, and SMR superfamilies take advantage of the motive force provided by H^+^ or Na^+^ ion gradients to obtain the energy to extrude various compounds [16,17,18,19,20], the ABC pumps function by the hydrolysis of ATP to transport their substrates across bacterial membranes [21,22]. In addition, the structural composition of efflux pumps is different among the superfamilies (Figure 2).

### 1.1. MATE Efflux Pumps

Members of the MATE family consist of twelve α-helical transmembrane helices (TMHs) exploiting electrochemical ion gradients to drive the efflux of cationic and polyaromatic drugs [23]. Structurally, MATE pumps have a V-shaped central cavity facing toward the extracellular space located halfway along the lipidic bilayer by maintaining an outward-open state [14]. The principal substrates recognized by MATE transporters are fluoroquinolones [19]. NorM is, for example, a MATE efflux pump that protects pathogens from the damage induced by reactive oxygen species and exports antimicrobial cationic compounds or antibiotics, as demonstrated in *Neisseria gonorrhoeae* [24,25,26]. NorM interaction with substrates depends on ionic and hydrogen bonds with the involvement of some conserved amino acid residues [25]. The drug-binding pocket is near the periplasmic side and does not function in an inward-open state, pointing out the importance of the conformational state of this pump [14]. Moreover, DinF (DNA damage-inducible protein F), another MATE member, confers pneumococci resistance to moxifloxacin, ciprofloxacin, and levofloxacin [27].

### 1.2. SMR Efflux Pumps

The SMR family members are composed of four trans-membrane helices (TMHs) spanning the cytoplasmic membrane, and they function as homodimers or heterodimers [28]. While three TMHs form a substrate-binding chamber, the fourth one contributes to interactions involved in the dimerization process of the pump [28,29]. This transport system alternates between inward and outward states following conformational changes that happen in opposite orientations in the membrane [14]. The SMR pumps promote the solubilization of a broad range of drugs such as disinfecting agents, toxic lipophilic molecules, or toxic metabolites [28,29]. Genes encoding SMR proteins can be horizontally transferred through plasmids or mobile genetic elements by conferring multidrug resistance [29].

### 1.3. ABC Efflux Pumps

ABC transporters are composed of trans-membrane domains (TMDs) containing substrate-binding pockets and nucleotide-binding domains (NBDs) that bind and hydrolyze ATP to promote substrate transport [30,31,32]. The translocation of substrates across the cell membrane is due to conformational changes happening by NBD dimerization and dissociation, a process mediated by ATP. In this way, an ABC transporter alternates an inward-open and outward-open conformation [33,34,35]. ABC transporters can be homodimeric and heterodimeric but, sometimes, they can associate with an outer membrane protein, increasing bacterial virulence and drug resistance. The tripartite complex MacAB-TolC efflux pump is, for example, a widely studied ABC-type transporter in Gram-negative bacteria [36]. This complex is composed of the inner membrane protein MacB, the periplasmic protein MacA, and the outer membrane protein TolC. While the protein MacB contains a C-terminal cytoplasmic tail and an N-terminal domain involved in the binding to ATP, the protein MacA binds to the lipopolysaccharide core and is activated by ATPase [37,38]. It has been shown that the absence of the MacAB efflux pump makes *Serratia marcescens* susceptible to aminoglycosides and polymyxins or that MacAB confers resistance to antibiotics in *Agrobacterium tumefaciens* [39,40]. Various examples of ABC transporters have been described in Gram-positive microorganisms such as LmrA in *Lactococcus lactis*, EfrAB in *Enterococcus faecalis,* or PatA/B in *Streptococcus pneumoniae*. LmrA recognizes and transports macrolides and lincosamides [41,42], EfrAB primarily extrudes gentamicin, streptomycin, and chloramphenicol [43], while PatA/B promotes defense against ciprofloxacin and norfloxacin [44].

### 1.4. PACE Efflux Pumps

PACE transporters are encoded by highly conserved genes among bacterial species and contribute to the extrusion of biocides, such as chlorhexidine [45]. The PACE family transporters share similar sizes and secondary structures with SMR transporters. The best-described PACE transporter is the Acinetobacter chrlorhexidine efflux protein I (or AceI) in *Acinetobacter baumannii* [46]. The structure of AceI depends on the pH and is the result of an equilibrium between a monomeric and dimeric form. Glutamic acid residue was revealed to be useful for its dimerization [47]. Moreover, AceI transcription has been shown to be augmented in the presence of increasing levels of chlorhexidine that binds to the transcriptional protein AceR, allowing bacteria to extrude chlorhexidine itself [47]. Recently, a new PACE transporter, named PA2889, has been characterized in *Pseudomonas aeruginosa*. Similar to AceI, PA2880 transports chlorhexidine and forms dimers in a solution, regardless of pH [48].

### 1.5. MFS Efflux Pumps

Compared to the other transporters, those belonging to the MFS family are the most characterized and show 12–14 TMHs, organized as two main domains, each of them consisting of bundles of six helices [23]. In this family of transporters, it is possible to distinguish three subgroups: (i) uniporters that transport the substrate across the lipid bilayer without using ions; (ii) symporters that transfer the substrate and ions in the same direction; and (iii) antiporters that favor the translocation of the substrate and ions but in opposite directions [14]. Similar to ABC transporters, but under the control of the electrochemical proton gradients, the two domains of MFS pumps alternate between inward-open and outward-open conformational states during a transport cycle. MFS pumps that are associated with multidrug resistance can be further identified as Drug: H^+^ antiporters 1 (DHA1) and 2 (DHA2) based on the conformation of their crystal structure. Some DHA1 transporters have been, for example, characterized by their specific conformation (i.e., YajR, EmrD, and MdfA in *Escherichia coli*), revealing the main helices that are preferentially targeted by drugs [49,50,51]. Moreover, several studies focused on the differences, among MFS members, in terms of drug–proton exchanges [52,53,54]. While members of DHA1 (i.e., MdfA) exchange a single H^+^ ion with a single substrate by favoring the transport of neutral or monovalent cationic drugs, members of DHA2 transfer two H^+^ ions for each substrate, regardless of its charge [14]. The extrusion of hydrophilic fluoroquinolones is, for example, mediated by the pump Lde in *Listeria monocytogenes* and NorA in *Staphylococcus aureus*, while macrolides are eliminated by the pump Mef in *S. pneumoniae* [55,56,57]. Moreover, MFS transporters may play relevant roles in other biological processes, such as microbial pathogenesis. For example, *A. baumannii* uses the AbaQ MFS transporter for its motility, while *S. aureus* uses the Tet38 MFS efflux pump in the adherence, internalization, and trafficking processes both in epithelial cells and in phagolysosomes [58,59,60].

### 1.6. RND Efflux Pumps

The RND pumps are composed of an outer membrane protein (OMP), an inner membrane protein (IMP), and a periplasmic adapter protein (PAP) that mediates connections between the OMP and IMP [20]. The TMDs of the RND transporters contain an internal structural repeat that facilitates proton movement, whereas the folding of the periplasmic domain can differ among the RND homologs. The site where the drug enters is called the pore domain and consists of four subdomains (PN1, PN2, PC1, and PC2). Altogether, these subdomains form two cavities responsible for drug recognition: the proximal and distal pockets. Both pockets have specific amino acids that confer to each of them the ability to specifically bind to a particular substrate rather than another one [14,61,62,63,64]. Examples of RND efflux pumps are AcrAB-TolC in *E. coli*, AdeABC in *A. baumannii*, MexAB-OprM in *P. aeruginosa,* and SmeABC in *Stenotrophomonas maltophilia* [65,66,67,68]. The RND efflux pumps are extensively involved in the elimination of huge categories of antibiotics and toxic compounds. RND transporters play, for example, an important role in mediating bacterial resistance to antibiotics and heavy metals. Furthermore, a mutation in specific amino acid residues in strategic domains of these efflux pumps may represent a mechanism to reduce or inhibit efflux pump affinity for their usual substrates, enhancing MDR [69].

## 2. Efflux Pump Genetic Regulation

Expression of the different efflux pumps is under the control of numerous regulatory pathways that seem to be triggered once the intracellular concentrations of toxic compounds reach high levels [70]. There are two main pathways described in terms of efflux pump genetic regulation: (i) the regulation mediated by transcriptional activators or repressors with DNA-binding motifs and (ii) the activation of two-component systems (TCS) that differently regulate gene expression. TetR is, for instance, a transcriptional regulator that controls the expression of the efflux-linked *tet* genes, which, in turn, confer resistance to tetracyclines, while the repressor FepR regulates the expression of the MATE efflux pump FepA, which makes *L. monocytogenes* resistant to fluoroquinolones [71,72]. TCS are, instead, composed of a sensor histidine protein kinase (HK) and a cognate response regulator (RR) [73,74]. The structure of an HK is composed of four domains: a sensor domain, an intracellular transduction domain, a cytoplasmic sensor domain, and a well-conserved intracellular kinase domain responsible for HK autophosphorylation and the subsequent transfer of a phosphoryl group to the RR. The sensor domains of HK can be intramembrane or placed on the cell surface and can have several structural foldings [75,76]. The structure of a RR is attributable to two main domains, indicated as the receiver domain and the effector domain. The receiver domain contains conserved aspartate residues that are commonly phosphorylated by the HK, whereas the effector domain has DNA-binding properties that are useful for its interaction with target sequences in the bacterial genome in order to regulate their expression [77,78,79]. TCS can be activated by different environmental signals, such as host receptors, antimicrobials, bivalent ions, organic solvents, pH, and oxidative stress. For several pathogens, a strict association between TCS and antibiotic resistance has been described [80,81]. In light of this, the main goal of this review is to sum up all information about the importance of TCS in regulating multidrug efflux pump expression, one of the better-identified mechanisms of antibiotic resistance. In Table 1, examples of TCS-mediated antibiotic resistance, including those mediated by mechanisms other than efflux pumps, are reported for the pathogens cited in this paper. The multifactorial roles played by TCS on MDR should be taken into consideration when developing targeted therapies against infectious diseases.

## 3. Gram-Negative Bacteria

### 3.1. Acinetobacter baumannii

*Acinetobacter baumannii* (phylum: *Proteobacteria*; class: *Gammaproteobacteria*; order: *Pseudomonadales*; family: *Moraxeaceae*) is a Gram-negative bacterium that is responsible for a variety of diseases such as bacteremia, meningitis, urinary tract infections, pneumonia, soft tissue, and skin infections [112]. Among the multidrug-resistant pathogens, *A. baumannii* behaves as an opportunistic microorganism by playing a significant role in hospital-acquired infections [113]. Epidemiological estimations revealed that carbapenem-resistant *A. baumannii* strains are isolated in a high percentage of intensive care unit patients and can cause serious infections [114,115]. Due to its ability to resist multiple antimicrobials, *A. baumannii* has been included among the most serious ESKAPE pathogens (*E. faecium*, *S. aureus*, *K. pneumoniae*, *A. baumannii*, *P. aeruginosa,* and *Enterobacter* species) [116,117,118]. As an MDR microorganism, *A. baumannii* has the capacity of using several mechanisms to escape antibiotic-mediated killing, including the production of modifying enzymes (e.g., β-lactamases), the alteration of membrane permeability, target modification, and the expression of multidrug efflux pumps [119,120,121]. 

To date, among the efflux transporter families, three RND efflux pumps have been fully described and associated with intrinsic antibiotic resistance in *A. baumannii*: AdeFGH, AdeIJK, and AdeABC [122,123,124,125,126]. Their expression is tightly regulated by transcriptional regulators. For example, the expression of the AdeFGH pump is controlled by the LysR-type regulator AdeL, while AdeIJK is by the TetR family transcriptional regulator AdeN [119,127]. The RND-type efflux pump AdeABC has been greatly studied in *A. baumannii* pathogenesis and is linked to multidrug resistance. The AdeB protein contains twelve transmembrane domains, whereas AdeC is similar to the outer membrane protein OmprM from *P. aeruginosa*. The AdeABC efflux pump confers resistance to various classes of antibiotics such as aminoglycosides, tigecycline, gentamicin, and fluoroquinolones [128]. The genes encoding for the three elements of this efflux pump (namely *adeA*, *adeB,* and *adeC*) are contiguous, co-transcribed, and preceded by two genes, which encode the AdeR and AdeS (a response regulator and a histidine kinase, respectively) and are transcribed in the opposite direction [129]. The absence of AdeR and AdeS renders *A. baumannii* susceptible to aminoglycosides or phleomycin [129,130], while *adeAB* transcription is undetectable in susceptible strains. On the contrary, analyses of spontaneous gentamicin-resistant *A. baumannii* mutants revealed major expression of *adeAB* genes. Moreover, the presence of specific mutations in the sensor kinase domain and in the response regulator (Thr153→Met and Pro116→Leu, respectively) promotes constitutive resistance. These point mutations reside in important regions of the components of the AdeRS regulatory system. The Thr153→Met substitution is close to the putative amino acid residue of autophosphorylation and is seemingly involved in the inhibition of the phosphorylase activity of AdeS, whereas the Pro116→Met mutation involves the output domain of the response regulator and enhances AdeR affinity for its DNA sequence target [129]. Several observations have been performed in clinical isolates of *A. baumannii,* revealing how functional mutations were mainly present in the conserved domains of the AdeRS TCS and were associated with the overexpression of the AdeABC pump and the loss of antibiotic susceptibility [131,132,133,134]. Some insertional sequences in *adeS* have been further described in *A. baumannii* and associated with overexpression of the AdeABC efflux pump and decreased susceptibility to tigecycline due to constitutive activation of AdeR. By analyzing the *adeR* sequences of clinical tigecycline resistant *A. baumannii* isolates, some researchers noted the presence of point mutations in the DNA-binding domain of the response regulator of bacteria, showing a high minimal inhibitory concentration (MIC) of tigecycline. This can be referred to as the AdeR reduced binding affinity to the intercistronic spacer, thus enabling a major expression of the efflux pump and the consequent raising of MIC values of tigecycline [135]. 

An RND efflux pump is typically formed by three proteins that together render the whole complex functional. Interestingly, the *adeC* gene is not always present in all the *A. baumannii* strains but, despite this, they continue to show antibiotic resistance, suggesting that the complex AdeAB can function alone. In the ATCC 17978 strain, naturally devoid of *adeC*, it has been shown that the deletion of *adeRS* determined susceptibility to a limited group of antimicrobial substances, such as chlorhexidine and pentamidine, and that resistance to these dicationic compounds was due to the AdeRS-dependent expression of *adeAB* in ATCC 17978 [136]. However, rather than resistance to antimicrobial substances, the overexpression of AdeABC is particularly linked to increased virulence in vivo justifying, in this way, the onset of so many single-point mutations in the AdeRS TCS. The AdeRS TCS is, indeed, involved in biofilm formation, epithelial cell killing, and the regulation of genes related to motility, DNA uptake channels, and virulence in a *Galleria mellonella* infection model [136,137].

In *A. baumannii,* the BaeSR TCS has also been found to be involved in antibiotic-resistant phenotypes and was proposed as a regulator of several efflux pumps: AdeABC, AdeIJK, and MacAB-TolC. Some authors showed that the BaeSR TCS responds to high osmotic stress and influences the susceptibility of *A. baumannii* to tigecycline by the regulation of *adeA* and *adeB* expression [138]. Furthermore, *baeR* deletion determines a reduction, in terms of expression, of AdeIJK and MacAB-TolC levels, whereas tannic acid, which shows potential properties as an antibiotic adjuvant, induces gene expression of the AdeABC, AdeIJK, and MacAB-TolC efflux pumps [139]. Despite these phenotypical characterizations, no protein DNA complexes have been observed between recombinant BaeR and *adeA*, *adeI,* and *macA* promoters.

### 3.2. Escherichia coli

*Escherichia coli* (phylum: *Pseudomonadota*; class: *Gammaproteobacteria*; order: *Enterobacteriales*; family: *Enterobacteriaceae*) is a significant component of the human microbiota, but it can cause intestinal or extra-intestinal infections and represents a great reservoir of resistance genes [140]. A large spectrum of virulence factors has been investigated and associated with specific *E. coli* phenotypes [141]. Several papers focused on the role played by TCS in multidrug efflux pump expression in *E. coli*. The EvgA/EvgS TCS of *E. coli* shows high structural similarity to the BvgA/BvgS TCS of *Bordetella pertussis* [142]. The N-terminal region of the sensor EvgS is localized in the periplasm, while the C-terminal one is cytoplasmic and contains the typical four domains of an HK. The EvgS protein responds to environmental stimuli through its periplasmic domain and transduces signals into the response regulator EvgA via a series of phosphorylation reactions. Mutations in the linker region of EvgS have been described and associated with constitutive activation of this TCS that induces *emrKY* expression, an MFS-type exporter, and confers resistance to sodium deoxycholate [143]. The linkage between *emrKY* and multidrug antibiotic resistance has been confirmed by using several drugs and toxic compounds, such as doxorubicin, novobiocin, crystal violet, rhodamine 6G, and others, showing that the EvgA regulator is mainly responsible for the MDR mediated by the whole TCS [144]. Afterward, the relevance of EvgA/EvgS TCS in *E. coli* antibiotic resistance has been confirmed and extended by revealing that the EvgA promotes the expression of another MDR RND-type efflux pump, called YhiUV, and confirming the requirement of a phosphorylated response regulator [144]. Furthermore, in the presence of a deletion in the *yhiUV* gene, *evgA* up-regulation does not determine resistance to oxacillin, cloxacillin, or nafcillin, confirming the strict correlation between drug resistance, YhiUV, and EvgA overexpression [145]. Additionally, several authors showed that the expression of different drug efflux genes is controlled by a multifaceted regulatory network that involves both phosphorylated EvgA as the leading regulator and the Mg^2+^-responsive PhoPQ TCS, which cooperates with the activated EvgS sensor to promote expression of the outer-membrane channel TolC [146].

In two papers, published at almost the same time, the expression of the efflux pump MdtABC has been attributed to the overexpression of the BaeR response regulator. The MdtABC is an RND-type drug efflux transporter composed of three subunits that reduce bacterial susceptibility to novobiocin and bile salt derivates. In addition, BaeR confers low-level resistance to carbenicillin, aztreonam, carumonam, cefamandole, ceftazidime, cefmetazole, cefuroxime, and cefotaxime [145]. In contrast to the organization of the genetic loci of the EvgAS TCS and *emrKY*, the *baeSR* genes are located downstream of the *mdtABC* genes and are not transcribed in the opposite direction [147,148]. 

*E. coli K-12* has at least 20 MDR efflux pump genes that confer resistance, once overexpressed [149]. In this strain, several genes associated with metal-induced modifications of the membrane structure and functions have been described as regulated by the BasSR TCS. Moreover, *basR* mutation confers *E. coli* resistance to polymyxin B and sensitivity to deoxycholic acid. Deletion of *basSR* genes causes a loss of resistance to several antimicrobial agents (such as norfloxacin, tetracycline, erythromycin, and others) in *E. coli* and reduces the expression of the multidrug efflux pump *emrD* gene since BasR directly binds to its promoter [150].

### 3.3. Pseudomonas aeruginosa and Pseudomonas fluorescens 

*Pseudomonas aeruginosa* (phylum: *Pseudomonadota*; class: *Gammaproteobacteria*; order: *Pseudomonadales*; family: *Pseudomonadaceae*) is a Gram-negative bacillus representing one of the main causes of nosocomial infections, including ventilator-associated pneumonia (VAP) and surgical, bloodstream and urinary tract infections (UTIs) [151]. It causes also high mortality in people affected by cystic fibrosis, severe burns, or cancer. *P. aeruginosa* has the ability to evade immune responses by producing numerous virulence factors, adhering to the host surfaces and assembling biofilms [151,152,153]. The ability to form biofilms, together with low membrane permeability and intrinsic mechanisms of resistance, makes *P. aeruginosa* less susceptible to antibiotics [151,154,155]. Particularly, the overexpression of multidrug efflux pumps in *P. aeruginosa* is associated with the ineffectiveness of anti-pseudomonal drugs. Examples of well-characterized efflux pumps in *P. aeruginosa* are the RND superfamily members MexAB-OprM, MexCD-OprJ, and MexEF-OprN. MexAB-OprM expression is controlled by the repressor genes *mexR*, *nalC,* and *nalD* and is constitutively expressed in *P. aeruginosa* strains [156,157,158]. Mutations found in these genes are associated with MexAB-OprM overexpression and MDR in both lab and clinical isolates [159,160,161,162]. MexAB-OprM can pump several antibiotics, such as quinolones, macrolides, and chloramphenicol, and is highly expressed in carbapenemase-producing *P. aeruginosa* strains [163,164]. MexCD-OprJ is, instead, associated with resistance to fluoroquinolones, macrolides, novobiocin, tetracyclines, chloramphenicol, and zwitterionic cephalosporins, and its expression is mainly regulated by the repressor *nfxB* [163,165,166,167,168,169,170]. MexEF-OprN expression is regulated by MexT, a LysR-like transcriptional regulator, and MexS [171,172]. Overexpression of this efflux pump arises principally following the onset of revertant mutations that activate MexT, since inactivating *mexT* mutations prevail in wild type *P. aeruginosa* strains [173]. The presence of mutations in *mexS* has been also observed in clinical isolates and linked to the overexpression of MexEF-OprN [174,175,176]. In the context of biofilm assembly, efflux pumps play a substantial role in *P. aeruginosa* pathogenesis for different reasons: (i) several molecules are transported to the extracellular environment in the course of biofilm formation; (2) specific substances are used in quorum sensing signaling pathways mediating intercellular communications; and (3) antibiotics are expelled by efflux pumps, contributing to the preservation of the microbial community [177,178,179]. The MexAB-OprM efflux pump, for example, delivers acyl-homoserine lactones (AHL) to the extracellular compartments, whereas the MexEF-OprN efflux pump promotes the outflow of 4-hydroxy-2-heptylquinoline in *P. aeruginosa* quorum sensing [177,180].

The MexXY-OprM pump is known to confer resistance to the aminoglycosides, which are mainly chosen in the treatment of patients suffering from lung infections, such as cystic fibrosis [181,182]. The *mexX* and *mexY* genes are part of the same operon under the control of the repressor MexZ, while the OprM protein is encoded by another gene that belongs to *mexAB-oprM*, a multidrug efflux pump, as mentioned above [183,184,185]. Expression of the *mexXY* operon is induced by antimicrobials targeting the ribosomes or following alterations in the translational machinery [186,187,188]. The AmgRS TCS has been linked to the acquisition of aminoglycoside resistance in *P. aeruginosa* by *mexXY* overexpression as a result of gain-of-function mutations in the *amgS* sensor kinase gene and exposure to aminoglycosides themselves, resulting in protection from membrane damage [189]. Similar observations were made for *mexAB-oprM* overexpression, underlining the fact that membrane-perturbing agents, such as diamide, favor the AmgRS-dependent *mexAB-oprM* expression, even though this efflux pump does not seem to play a role in resistance to aminoglycosides and diamide. In light of this, the authors speculate on the possibility that aminoglycosides can induce the expression of OprM, an outer membrane protein that primarily cooperates with the MexXY pump, which, instead, contributes to aminoglycoside resistance since the *oprM* gene has its own promoter and can be transcribed independently from *mexAB* [190]. Furthermore, a comparative genomic analysis has highlighted the direct role played by the CpxRS TCS in mediating *mexAB-oprM* expression and the consequent multidrug resistance in the *mexR*-defective *nalB*-type *P. aeruginosa* strain with a phenotype detectable in clinical isolates during antibiotic therapy [191].

Strains isolated from patients with cystic fibrosis often exhibit mutations in the *mexZ* gene resulting in marked *mexXY* expression [184,192,193]. Moreover, clinical *P. aeruginosa* isolates overexpressing MexXY with an intact *mexZ* gene have sometimes been reported, suggesting additional regulatory pathways [181,193]. A genomic analysis of polymorphisms revealed the presence of a point mutation in the receiver domain of the response regulator ParR that is part of the ParRS TCS (standing for peptide-adaptive resistance regulator and sensor). The ParRS TCS has been described as a regulatory system responsible for the *P. aeruginosa* adaptive resistance to polycationic peptides, such as colistin, by the lipopolysaccharide (LPS) modification operon *arnBCADTEF-ugd* [194]. Inactivation of *parS* and *paRS* genes deletes multidrug resistance of the tested strains in the presence of colistin, and a single mutation in the first transmembrane domain of the sensor ParS confers an MDR phenotype due to an up-regulation of the *mexY* gene and down-regulation of the OprD porin. Even in the case of this TCS, several nucleotide substitutions in *parS* or in *parR* have been found in *P. aeruginosa* clinical isolates [195]. 

Few ABC transporters are known for *P. aeruginosa*. In a paper, several authors showed that the PA4456-4451 efflux pump increases bacterial resistance to tetracycline and that the PhoQP TCS negatively controls its expression in a wild type strain under low Mg^2+^ conditions, suggesting its role in *P. aeruginosa* intrinsic resistance [82].

Another efflux pump, called CzcCBA, has been associated with *P. aeruginosa* resistance to heavy metals such as zinc, cadmium, and cobalt [196,197]. A central role in *czcCBA* expression has been attributed to CzcRS (standing for cobalt/zinc/cadmum regulator and sensor) TCS, whose transcription is further enhanced upon exposure to heavy metals. The same resistance profile has been found for carbapenem-resistant strains that show a reduced quantity of the OprD protein. Interestingly, a single point mutation in the residue Val-194, in correspondence with the second transmembrane segment of the CzcS sensor, greatly contributes to a decrease in the levels of OprD expression, whereas it augments resistance to imipenem [197]. On the contrary, the overexpression of the CzcR response regulator does not determine a major resistance to heavy metals, rendering bacteria more susceptible to them because it does not up-regulate the *oprD* gene, suggesting that this type of regulation does not require the phosphorylation of CzcR compared to the role played by the phosphorylated CzcR in triggering the transcription of the CzcCBA [197]. In light of these evaluations, the authors remark on the possibility that both the overexpression of the CzcCBA efflux pump and the down-regulation of the OprD porin can represent defense mechanisms to avoid the increase in dangerous molecules inside the bacterial cell [197]. Furthermore, the Zinc Uptake Regulator (also known as Zur protein) has been shown to be involved in the control of intracellular zinc by the activation of CzcR. Indeed, in the presence of high quantities of Zn inside the bacterial cell, Zur dimerizes with zinc and enhances the expression of the regulator CzcR that, in turn, promotes the up-regulation of the zinc-extruding efflux pump CzcCBA [198]. CzcRS-dependent zinc induction plays a relevant role in *P. aeruginosa* since urine can release zinc upon contact with siliconized latex urinary catheters favoring carbepenem-resistance mediated by OprD expression [199,200]. These observations are in line with those performed with the TCS of uropathogens during the onset and progression of urinary tract infections [201].

Additionally, in *P. fluorescens*, the RND superfamily efflux pump EmhABS has been found to induce resistance to antibiotics such as ampicillin, chloramphenicol, and tetracycline [202,203]. Antibiotic resistance associated with the RstA/RstB TCS has been linked to increased expression of EmhABS and the MexCD-OprJ efflux pump, as demonstrated by the reduction in transcriptional levels of *emhA* and *mexC* after deletion of the *rstA* gene [204]. Similar to other studies performed on TCS regulation, even in this case, direct binding of the response regulator RstA to the *emhA* promoter was shown, underlying the role of the conserved aspartate residue 52 (D52) in the phosphorylation process since the substitution of this amino acid residue with a non-functional alanine residue increased bacterial susceptibility to numerous antibiotics such as chloramphenicol, gentamicin, kanamycin, and lomefloxacin [204]. Deletion of the sensor domain RstB did not have any effect in terms of antibiotic resistance. It seems that TCS RstA/RstB can function as a sensor for nitrosative stress, as suggested by the up-regulation of pyoverdine biosynthesis [204].

### 3.4. Salmonella enterica

*Salmonella enterica* (phylum: *Pseudomonadota*; class: *Gammaproteobacteria*; order: *Enterobacteriales*; family: *Enterobacteriaceae*) is a Gram-negative bacterium associated with foodborne disease and is the cause of Salmonellosis. It is the main cause of diarrhea, and more than 500 million humans are annually infected. After gaining access to the host, *Salmonella* overcomes the acid defenses of the stomach causing damage to the gastrointestinal tract [205]. For example, *S. enterica* behaves as an intracellular pathogen because of its capacity to enter the M cells, and it consequently crosses the epithelial barrier of the gut, causing a variety of diseases ranging from gastroenteritis to typhoid fever [206]. The host cells that are infected by *Salmonella* undergo cytoskeletal alterations that facilitate *Salmonella* internalization. This is made possible thanks to the expression of different Pathogenicity Islands (PIs) by several virulent *Salmonella* strains. PIs, for example, promote the production of a type of secretion system (T3SS) that mediates the injection of effector proteins inside the host cells with the effect of facilitating the invasion of epithelial cells and intracellular survival [207]. Moreover, *Salmonella* species invade the gallbladder, form biofilms in gallstones, and may cause irritable bowel syndrome and reinfection of the gastrointestinal tract [208,209].

Nowadays, multidrug resistance is increasingly becoming a relevant problem in the case of *Salmonella* species, and several clinical isolates were found to be resistant to fluoroquinolones [210,211,212,213,214,215]. Ceftriaxone and azithromycin currently represent the secondary drugs of choice for treating patients suffering from typhus when fluoroquinolone-resistant strains are detected. Furthermore, although carbapenems and colistin are taken into consideration as antimicrobial agents against multidrug-resistant *Salmonella* isolates, cases of carbapenemase- and colistin-resistance phenotypes are beginning to prevail. Even for this pathogen, efflux pumps represent an important mechanism to subvert the anti-bacterial effects mediated by drugs [216]. The RND efflux pump AcrAB-TolC is, for instance, overexpressed during *S. enterica* exposure to antimicrobial agents through a series of transcriptional activators [217]. Moreover, in *S. enterica* serovar *typhimurium*, the MATE type efflux pump MdtK was found to be responsible for extruding drugs, such as aminoglycosides and fluoroquinolones, from the bacterial cell [218]. Among the MFS-type efflux transporters in *S. enterica*, the EmrAB is an important pump because it has been reported as a way to develop resistance to different antimicrobial drugs such as novobiocin, sodium deoxycholate, and nalidixic acid [219]. 

Several efflux pumps have been discovered in *S. enterica* serovar *typhimurium,* and each of them can confer specific antibiotic resistance. For example, the overexpression of the transporter MdsABC determines resistance to novobiocin, acriflavine, crystal violet, and other antimicrobial agents, while the up-regulation of the efflux pump *emrAB* genes does not render bacteria susceptible to novobiocin, nalidixic acid, or rhodamine 6G [220]. Remarkably, the overexpression of *macAB* drug exporter genes is induced by the PhoP-PhoQ TCS, which is known for preserving bacteria from bile salts [221]. Specifically, this TCS has been identified as a direct repressor of *macAB* genes since the latter are down-regulated in a *phoP* mutant compared to the wild type strain. Moreover, Mg^2+^ ions have been proposed as the main signal activating the PhoPQ TCS that, in turn, enhances the expression of *macAB* ABC-transporter genes [220]. Transposon mutagenesis experiments revealed the role played by the BaeRS TCS in the acquisition of *S. enterica* resistance to antibiotics, showing that the response regulator BaeR confers more resistance to broad-spectrum cephalosporins (i.e., ceftriaxone) than narrow-spectrum and expanded-spectrum drugs (i.e., cephalothim and cefamandole, respectively). Moreover, the expression of BaeR regulates the synthesis of two outer membrane proteins, named OmpW and STM3031 [222]. In another paper, it has been reported that the multidrug efflux pump *mdtABC* and *acrD* genes are positively regulated by the BaeSR TCS. The overexpression of *baeR* increases *S. enterica* serovar *typhimurium* resistance to several β-lactams, such as oxacillin, cloxacillin, and nafcillin, an effect that is also dependent on the expression of the TolC exporter, as demonstrated by using mutant strains for the TCS and/or the components of the efflux pumps [223]. In addition, copper and zinc have been indicated as signals capable of triggering the activation of the BaeSR TCS for the induction of *mdtA* and *acrD* gene expression, contributing to metal adaptation and resistance in *S. enterica* [223]. *S. enterica* resistance to aminoglycosides and β-lactams in both susceptible and clinical isolates has been studied in the context of CpxAR TCS activation. The absence of *cpxR* decreases the MICs of gentamicin, apramycin, neomycin, ceftriaxone, amikacin, ceftiofur, and cefquinome, compared to the wild type strains [224]. Moreover, complementation of a Δ*cpxR S. enterica* serovar *typhimurium* strain with a plasmid encoding for CpxR significantly reduces the expression of various porins (i.e OmpF, OmpC, OmpD, and OmpW) and up-regulates STM301 and STM1530 proteins in association with ceftriaxone [224]. A high incidence of antibiotic resistance has been further reported for *S. enterica* serovar Enteritidis isolates with the ACSSuT resistance profile (standing for resistance to ampicillin, chloramphenicol, streptomycin, sulfamethoxazole, and tetracycline) [225,226]. The analysis of several TCS showed that the absence of PhoP and CpxR response regulators increases susceptibility to cephalosporins and quinolones by improving the expression of membrane porins (OmpC, OmpD, and OmpF) and by reducing efflux pump genes, such as *acrA*, *macB*, and *mdtK* [227].

### 3.5. Stenotrophomonas maltophilia

*Stenotrophomonas maltophilia* (phylum: *Pseudomonadota*; class: *Gammaproteobacteria*; order: *Xanthomonadales*; family: *Xanthomonadaceae*) is a Gram-negative opportunistic bacterium that shows high-level intrinsic resistance to several antimicrobial drugs [228]. Different RND-type efflux pumps have been identified in *S. maltophilia*: SmeABC, SmeDEF, SmeOP, SmeIJK, SmeVWX, and SmeYZ [229,230,231,232,233,234]. SmeDEF and SmeOP are under the control of the TetR-type transcriptional repressors, SmeT and smeRo, whereas the expression of the SmeVWX pump is regulated by a LysR-type transcription regulator [231,234,235]. Numerous antibiotics are extruded by these efflux pumps. While SmeDEF, for example, recognizes chloramphenicol, quinolone, tetracycline, and macrolides, SmeOP pumps out doxycycline, nalidixic acid, aminoglycosides and macrolides [229,234]. A sequence analysis of the genetic organization around the *smeYZ* genes revealed the presence of two genes encoding for an RR and HK that were named *smeRy* and *smeSy*, respectively. The deletion of the whole *smeRySy* TCS results in enhanced susceptibility to SmeYZ substrates and the reduction in *smeYZ* expression [236]. It is surprising that the inactivation of SmeRySy TCS increases the resistance to antimicrobial drugs that are not commonly recognized by the SmeYZ efflux pump but by the SmeDEF pump, whose operon is, instead, up-regulated in a strain deleted for this TCS [236]. In this case, *S. maltophilia* acquires resistance to chloramphenicol, ciprofloxacin, tetracycline, and macrolides. Furthermore, Δ*smeRy* bacteria show an increased susceptibility against aminoglycosides, which are substrates of SmeYZ, but do not exhibit susceptibility against the substrates of the SmeDEF efflux pump. Altogether, these evaluations highlight that the inverse expression of both pumps may cooperate to sustain bacterial survival [236].

Cationic antimicrobial polypeptides (CAPs) are frequently used to treat MDR bacteria, and it has been shown that the PhoQP TCS plays a role played in resistance against them in *S. maltophilia*. Under low concentrations of Mg^2+^, the response regulator PhoP mediates the expression of the SmeZ efflux transporter, determining aminoglycoside resistance. Moreover, bacteria devoid of the *phoP* gene show a major permeability in the cell membrane compared to wild type bacteria [237]. The MacABCsm is an ABC-type efflux pump that is able to extrude macrolides, aminoglycosides, and polymyxins [238]. Upstream of the *macABCsm* cluster, two genes encoding for the components of the MacRS TCS are placed. In contrast to other TCS, this MacRS TCS is divergently transcribed from the *macABCsm* operon, suggesting its critical role in the expression of this efflux pump [238]. The MacABCsm pump is different from the MacAB homologs of other microorganisms because it has its own outer membrane channel, is intrinsically expressed, and is involved in several physiological functions, such as oxidative stress tolerance and biofilm assembly [238].

### 3.6. Klebsiella pneumoniae

*Klebsiella pneumoniae* (phylum: *Proteobacteria*; class: *Gammaproteobacteria*; order: *Enterobacteriales*; family: *Enterobacteriaceae*) is a Gram-negative facultative bacterium belonging to the *Enterobacteriaceae* family. It causes severe diseases such as urinary tract infections, soft tissue infections, septicemia, and pneumonia [239]. The main reservoir for *K. pneumoniae* is the gastrointestinal tract, whose colonization depends on the capacity of this pathogen to interact with host surfaces, assemble biofilms, and survive against stressful responses [240,241,242]. *K. pneumoniae* is responsible for both nosocomial and community-acquired infections and is frequently isolated from liver abscesses [243,244,245,246]. For this microorganism, antimicrobial resistance was particularly linked to therapeutic failures of antibiotics such as quinolones, aminoglycosides, and β-lactams. Among TCS involved in sensing stressing responses, the CpxAR has been investigated in numerous bacteria, such as *E. coli* and *S. enterica* [247,248,249,250,251]. It has been demonstrated that the sensor kinase CpxA is induced by physical, chemical, and biological stresses, incorrectly folded proteins, detergents, etc. The hyper-virulent *K. pneumoniae* NTUH-K2044 strain has been used to investigate the role of the CpxAR in antimicrobial resistance [252]. The deletion of *cpxAR* in this strain resulted in sensitivity to cefepime, cefotaxime, ceftazidime, and chloramphenicol and in the relative inability to express efflux pump-encoding genes, such as *acrB*, *acrD,* and *eefB*, highlighting the important contribution of CpxR to MDR [252]. Interestingly, the deletion of the whole TCS enhances the expression of three proteins in the outer membrane, suggesting that proteins resembling *E. coli* porins are involved. A DNA-protein binding assay showed that CpxR binds near the gene encoding for a homolog of *E. coli* OmpC, called OmpC^KP^ (known also as *kpnO*) [252]. In another study, the role of the *kpnO* porin in MDR has been investigated in relation to its regulation by the PhoBR TCS. The deletion of *phoB* renders *K. pneumoniae* sensitive to antibiotics (i.e., ceftazidime, cefepime, ceftriazone, ertapenem, carbenicillin, and quinolones) by decreasing *kpnO* expression [253]. Tigecycline is currently one of the most frequently used drugs against carbapenem-resistant pathogens. Despite this, numerous carbapenem-resistant clinical strains are becoming more and more resistant to tigecycline [254,255,256]. For several microorganisms, resistance to this antimicrobial compound has been reported to be mediated by the overexpression and activity of RND transporters, such as the AcrAB-TolC efflux pumps [257]. Additionally, there are few papers focused on the correlation between antibiotic resistance and metal detoxification, suggesting that the presence of heavy metals can sometimes up-regulate resistance to antibiotics, such as β-lactams [258,259]. In *K. pneumoniae,* the CusSR TCS was responsive to copper and silver ions that induced the phosphorylation of the regulator CusR. This, in turn, promoted the positive regulation of CusCFBA efflux pumps, rendering bacteria resistant to the inducing ions. Based on these observations, the role of the CusSR TCS was further studied in carbapenem-resistant *K. pneumoniae* (CRKP) strains by showing that this TCS also regulates tigecycline susceptibility [260].

## 4. Gram-Positive Bacteria

### 4.1. Staphylococcus aureus

Staphylococci (phylum: *Firmicutes*; class: *Bacilli*; order: *Bacillales*; family: *Staphylo-coccaceae*) are Gram-positive bacteria and are considered commensals or opportunistic microorganisms [261]. Staphylococci are differentiated into two main groups: coagulase-negative (CoNS) and coagulase-positive staphylococci (CoPS) on the basis of their capacity to use the coagulase enzyme to favor plasma coagulation. CoNS are considered less pathogenic than CoPS because they have fewer virulence factors, even though they have been also observed, for example, in pets, wild animals, and poultry meat, rather than humans [262,263,264,265,266]. Among CoNS, *Staphylococcus epidermidis*, *Staphylococcus haemolyticus,* and *Staphylococcus saprophyticus* are mainly involved in human infections, particularly in people suffering from immune system deficiencies or with implantable medical devices, as well as in oncologic patients and newborns [267,268]. Several cases of antibiotic resistance have been reported for CoNS because they were found resistant to methicillin because of the expression of the *mecC* gene located on a mobile genetic element [269,270,271]. *S. epidermidis* is, indeed, considered an important reservoir of resistance genes and is responsible for their transfer to other staphylococci. Among CoPS, *S. aureus* is the main representative human pathogen responsible for a wide-ranging spectrum of frequent diseases such as superficial skin abscesses, endocarditis, toxic shock syndrome, pneumonia, meningitis, and septicemia [272,273,274]. Staphylococcal infections are becoming increasingly difficult to treat because of multidrug resistance against β-lactams, quinolones, and aminoglycosides. Furthermore, the diffusion of methicillin-resistant *S. aureus* (MRSA) strains is stimulating researchers to find novel therapeutic targets and technologies [273,275]. Efflux pumps in *S. aureus* represent a noteworthy theme because hospital-acquired *S. aureus* often uses this mechanism to acquire antibiotic resistance [276,277,278]. Different from CoNS, in *S. aureus*, various MDR efflux pumps have been characterized and found to be chromosomal or plasmid encoded. For instance, NorA and QacA belong to the MFS multidrug efflux family. NorA, together with NorB and NorC, confers resistance to hydrophilic fluoroquinolones, biocides, dyes, and chloramphenicol, and is expressed in a wide number of MRSA strains [279,280,281,282]. A plasmid-encoded efflux pump, the QacA transporter, is able to extrude a large spectrum of cationic lipophilic antimicrobial agents, such as diamidines and biguanidines [283,284,285]. 

As for other pathogens, TCS play important roles in the regulation of *S. aureus* responses to environmental stimuli, such as antibiotics. There are several examples regarding TCS that do not necessarily affect efflux pumps or transport systems. The WalKR TCS is, for example, highly conserved in Gram-positive bacteria and is involved in cell wall synthesis by regulating the expression of several gene encodings for autolysins and peptidoglycan hydrolases. Activation of this system results in the shortening of cell wall sugar chains and a reduction in the dense network of peptidoglycan cross bonds [286,287]. By modulating these processes, bacteria can reduce or suppress the effect of antibiotics acting on cell wall synthesis, such as vancomycin, which has D-alanyl-D-alanine as the main target [288]. Vancomycin-intermediate resistance in *S. aureus* strains (indicated as VISA) is associated with a high frequency of *walKR* mutations [287]. The single point A96T mutation affects WalKR TCS function by inhibiting the phosphorylation cascade and impairing cell wall modifications. This results, in turn, in decreased bacterial sensitivity to antimicrobial drugs [289]. Moreover, the histidine kinase of the WalKR TCS is physiologically inhibited by the protein YyccHI. A higher frequency of mutations in the *yyccHI* gene has been found in VISA clinical strains compared to vancomycin-sensitive *S. aureus* [290]. A similar mechanism has been described for the AirSR TCS, whose mutations down-regulate the expression of proteins involved in cell wall synthesis, thereby increasing resistance against vancomycin [291]. In addition, the VraRS TCS positively regulates the synthesis of several proteins promoting resistance to vancomycin [292]. The LytRS TCS has, instead, a double role because on the one hand, it regulates the secretion of extracellular DNA during biofilm formation, and on the other hand, it protects *S. aureus* from the action of CAPs. As far as the second aspect is concerned, the LytRS TCS acts when CAPs try to destroy the integrity of the cell membrane, leading to the death of bacteria through a series of lithic events. When the bacteria cell surface is altered by CAPs, the authophoshorylation of the sensor LytS is followed by the activation of the regulator LytR that increases the expression of the *irgAB* gene, which inhibits, in turn, programmed cell death [293,294].

Numerous studies focused on the mechanisms used by VISA strains to augment vancomycin resistance. These include increased peptidoglycan synthesis, decreased cell wall turnover, reduced cross-linking, decreased muropetide amidation and/or penicillin-binding protein levels, and efflux pumps [295,296,297,298,299]. Bacteria protect themselves not only from synthetic antibiotics but also from bacteriocins, antimicrobial molecules that are secreted by other bacteria to predominate other species. ATP-binding cassette (ABC) transporters are usually involved in bacterial self-immunity against secreted bacteriocins, and many gene encodings for ABC transporters are close to TCS genes [300,301]. In light of this, the GraRS TCS up-regulates the expression of the VraFG ATP-transporter, improving *S. aureus* resistance against both vancomycin and polymyxin B. Furthermore, alterations of the GraRS TCS have been found in association with increased bacterial lysis and negative surface charge, suggesting that this TCS might be a relevant target in the therapy of clinically resistant staphylococcal infections [302]. The BraS/BraR TCS has been investigated for its role in *S. aureus* resistance to bacitracin and nisin. “Bra” stands for bacitracin resistance-associated, where bacitracin is an antibiotic that inhibits peptidoglycan synthesis after binding to undecaprenyl pyrophosphate (UPP), the lipid carrier responsible for the translocation of cell envelope precursors to the extracellular side of the cell membrane [303]. Despite this, Gram-positive bacteria have developed resistance to this drug [304,305,306]. In *B. subtilis*, a TCS similar to BraS/BraR has been found to control the expression of an ABC transporter that confers resistance to bacitracin [305,307]. Structurally, BraS belongs to the intramembrane-sensing kinase subfamily because of the presence of a short sensing domain composed of two TMHs separated by a small peptide loop. This conformation seems to be involved in the cell envelope stress that is usually generated by antibiotics. Hiron A. et al. showed that absence of the whole BraS/BraR TCS makes staphylococci highly sensitive to bacitracin, and this regulatory system controls the synthesis of the BraDE and VraDE ABC transporters, which play different roles in antibiotic resistance. Unusually, while the BraDE transporter is required for activating the phosphorylated conformational state of BraR and has no relevant role in the antibiotic resistance, VraDE is sufficient to render bacteria resistant to bacitracin and nisin [308].

Based on their amino acid composition, bacteriocins can be classified into Class I and II. Class I bacteriocins, also known as lantibiotics, are further distinguished into type A and type B subgroups [309]. Among type A lantibiotics, nisin A plays an important role. Nisin A binds to lipid II, inhibits cell wall synthesis, and produces bactericidal effects by causing pore-formation [310,311], while Nukacin ISK I acts as a bacteriostatic agent by inhibiting cell wall synthesis [312]. The role played by GraRS, BraRS, and VraSR TCS in *S. aureus* has been investigated by observing the effects of TCS deletions in bacterial co-culture experiments [313]. Genetic inactivation in any of the three TCS causes increased susceptibility to nisin A and/or nukacin ISK I. BraRS TCS activity is associated with resistance to nisin A and nukacin ISK-1, whereas GraRS is involved in the regulation of cell surface charge. Indeed, the inactivation of *graRS* genes caused an increase in the attraction of the cationic peptides nisin A and nukacin ISK-1 to the cell surface. Lastly, the inactivation of the VraSR TCS led to an increase in *S. aureus* susceptibility to nukacin ISK-1, rather than nisin A [313]. In addition, nukacin ISK-1 and nisin A function as activating stimuli of the BraRS TCS in terms of *vraD* and *braA* gene up-expression, whereas *vraF* expression, under the GraRS control, is not induced by the same antibacterial agents [313]. 

Fosfomycin is another bactericidal antibiotic that behaves as a phosphoenolpyruvate (PEP) analog, which inhibits the protein MurA, altering the first steps in peptidoglycan biosynthesis [314,315]. Fosfomycin is useful for the treatment of numerous multidrug-resistant *S. aureus*-linked diseases, such as endocarditis [316]. An association between resistance to fosfomycin and the HptRS TCS has been found. This TCS is part of a novel hexose phosphate transport (HPT) system that includes the HptA protein (a putative phosphate sensor) and the UhpT protein (a hexose phosphate transporter). The authors suggest that HptA can sense extracellular phosphates and fosfomycin and activates HptRS that contribute to the expression of the UhpT protein that is not only the transporter for glucose-6-phosphate (G6P) but also fosfomycin, with the consequence that alterations involving HptA, HptRS, and/or UhpT can increase *S. aureus* resistance [317].

### 4.2. Streptococcus pneumoniae, Streptococcus agalactiae, and Streptococcus suis

*Streptococcus pneumoniae* (or pneumococcus) (phylum: *Firmicutes*; class: *Bacilli*; order: *Lactobacillales*; family: *Staphylococcaceae*) is a Gram-positive microorganism that colonizes the human nasopharynx [318]. However, it can become pathogenic and reach distant sites in the host, causing several types of infections such as otitis media, sinusitis, meningitis, community-acquired pneumonia, and septicemia [318]. The switch from a harmless commensal to a pathogen depends on pneumococcal dynamic interactions with the host, including the ability to evade immune responses [319,320,321,322,323,324]. All these pathogenic features are associated with the capacity of modulating the expression of virulence genes that are often under the control of numerous TCS [325]. The majority of TCS play important roles in pneumococcal virulence and a new one, the SirRH (standing for stress-induced response, also known as TCS01), has been recently characterized. In particular, the SirRH TCS has been shown to regulate the expression of *bceAB* genes that encode for an ABC transporter. Both the TCS and BceAB cooperate to provide resistance to a huge range of antimicrobial agents having, as molecular targets, the UPP or lipid II. Moreover, experimental data suggest that antimicrobial peptides can stimulate the ATPase activity of BceAB, inducing phosphorylation of the response regulator that, in turn, results in an up-regulation of *bceAB* genes [326]. *Streptococcus agalactiae* (group B streptococcus or GBS) is one of the main causes of neonatal death and clinical isolates are often associated with antibiotic resistance [327,328,329]. Numerous virulence factors facilitate host colonization, and the influx of neutrophils to GBS sites of infection, particularly, represents a fundamental step in the immune responses against this pathogen [330,331,332]. In GBS, a *bceRS* TCS operon has also been studied by demonstrating that antimicrobial peptides (AMPs), such as bacitracin, bind to the sensor BceS and enhance phosphorylation of the cognate regulator BceR, which results in an up-regulation of the transporter BceAB to pump out bacitracin and increase bacterial resistance, in association with an induced decrease in the negative charge of the bacterial cell membrane [73].

A comparative genomic analysis, performed in *Streptococcus suis*, has led several authors to find and characterize a novel bacitracin efflux pump that is under the control of the BceRS TCS [333]. Interestingly, the expression of the newly discovered SstFEG pump mediates tolerance to bacitracin independently of the BceAB transporter but is enhanced by the BceRS TCS. Furthermore, the deletion of *bceAB* determines the down-regulation of *bceRS*, which decreases SstFEG-mediated bacitracin resistance. Contrarily, the absence of *sstFEG* cannot regulate the BceAB and BceSR system in terms of bacitracin recognition [333]. This type of cross-activation between different TCS and efflux pumps has been also found in the genus *Bacillus*. In particular, it has been shown that high extracellular concentrations of bacitracin induce BceS to activate the non-cognate RR YvcP, which positively regulates the ABC transporter *yvcRS* operon involved in the subsequent detoxification of bacitracin [334]. Fluoroquinolones (FQs) are useful antibiotics for treating streptococcal infections and function by interacting with DNA gyrase and topoisomerase IV to inhibit bacterial replication. The abuse of these antibiotics has caused increased bacterial resistance to them, mainly due to single substitutions in the quinolone resistance-determining regions (QRSR) of gyrase and topoisomerase IV [335,336,337]. Moreover, the overexpression of efflux pumps can also confer fluoroquinolone resistance [338,339]. In the *S. suis* strain BB1013, the efflux pump SatAB has been, for example, linked to fluoroquinolone resistance, and its expression is regulated by the CiaRH TCS that, once deleted, significantly reduces susceptibility to norfloxacin and ciprofloxacin by means of the overexpression of SatA and SatB [340].

### 4.3. Listeria monocytogenes

*Listeria monocytogenes* (phylum: *Firmicutes*; class: *Bacilli*; order: *Bacillales*; family: *Listeriaceae*) is a facultative intracellular Gram-positive pathogen that can cause infection in humans after the ingestion of contaminated foods, leading to listeriosis. *L. monocytogenes* can reach distal organs, such as the placenta or the brain, causing abortion or meningoencephalitis [341]. The virulence pathways of *L. monocytogenes* are characterized by the expression of numerous genes involved in motility, host cell invasion, and intracellular replication [342,343]. The transcriptional control of virulence in *L. monocytogenes* has been described for several regulators such as PrfA, σ^B^, CodY, and VirR. While PrfA controls the expression of the pore-forming cytolysin listeriolysin (LLO) and the actin motility-inducing surface protein ActA, σ^B^ is interested in *L. monocytogenes* response to stress [344,345]. CodY is, instead, a transcriptional regulator the promotes the expression of PrfA and other genes when the quantity of branched-chain amino acids (BCACs) inside the host cells is low [346]. Interestingly, VirR is the response regulator of the VirRS TCS that has been studied in *L. monocytogenes* in terms of antibiotic resistance mediated by several systems of transport [347,348]. As described for the BceRS TCS in *Bacillus subtilis*, the sensor kinase VirS lacks an extracellular domain that can be able to recognize a particular ligand, but this property seems to be accomplished by an ABC transporter whose genes are usually placed close to the associated TCS genes. In this manner, the ABC transporter activates the sensor kinase, favoring the phosphorylation of the response regulator, thus forming a TCS/ABS transporter module [307,349,350]. Understanding this molecular crosstalk may be extremely useful in order to counteract antibiotic resistance even in the case of *L. monocytogenes,* which is becoming more nisin-resistant than other Gram-positive bacteria. It has been shown that VirR highly regulates the expression of the *dltABCD* operon that encodes the increase in the overall cell surface charge after incorporating D-alanine into lipoteichoic acid and controls the regulation of the protein MprF that protects *L. monocytogenes* from CAMP binding [351,352]. Moreover, the VirR regulates the production of an ATP-binding cassette (ABC) transporter, called AnrAB, whose role is involved in the detoxification of antimicrobial agents such as nisin, β-lactams, lantibiotics, and bacitracin [353]. A recently discovered ABC transporter, called VirAB, has been additionally associated with the VirR contribution to resistance against antimicrobial agents by showing that the deletion of *virR* and *virAB* significantly reduces the MIC of nisin, compared to the parental wild type *L. monocytogenes* strain. Furthermore, the VirR and AnrB proteins were found important for *L. monocytogenes* resistance to bacitracin, contrary to VirAB, suggesting that, under bacitracin stressing conditions, AnrAB may be involved in VirR signaling independently of the VirAB transporter. On the contrary, VirAB acts as the direct sensor of antimicrobial agents, such as nisin, potentially interacting with the VirS sensor kinase that, consequently, allows the expression of genes under the regulatory control of VirR [354]. The role played by the VirAB transporter has been further clarified in a recent paper by showing that in *L. monocytogenes* the VirSR TCS, in association to VirAB and AnrAB, creates a system that not only mediates nisin and bacitracin resistance, but also cephalosporins, ethidium bromide (EtBr), and benzalkonium chloride [355]. These conclusions were made after having seen that the deletion of *virAB* weakens the bacterial response to extracellular cefotaxim, suggesting that VirAB can modulate VirS HK activation. On the other hand, AnrAB is under the transcriptional control of VirAB and is involved in the detoxification process, even though the direction of drug transport is not completely clear. In addition, VirAB confers to *L. monocytogenes* kanamycin and tetracycline resistance, whereas the same feature is not detectable in VirSR and AnrAB main functions. In light of this, the VirAB-VirSR-AnrAB system mediates resistance to nisin, bacitracin, cephalosporins, EtBr, and BC by using VirAB for antimicrobial sensing and activation of VirSR and AnrAB for the transport of antimicrobials [355].

## 5. TCS Inhibitors: An Overview

**Figure 3:**
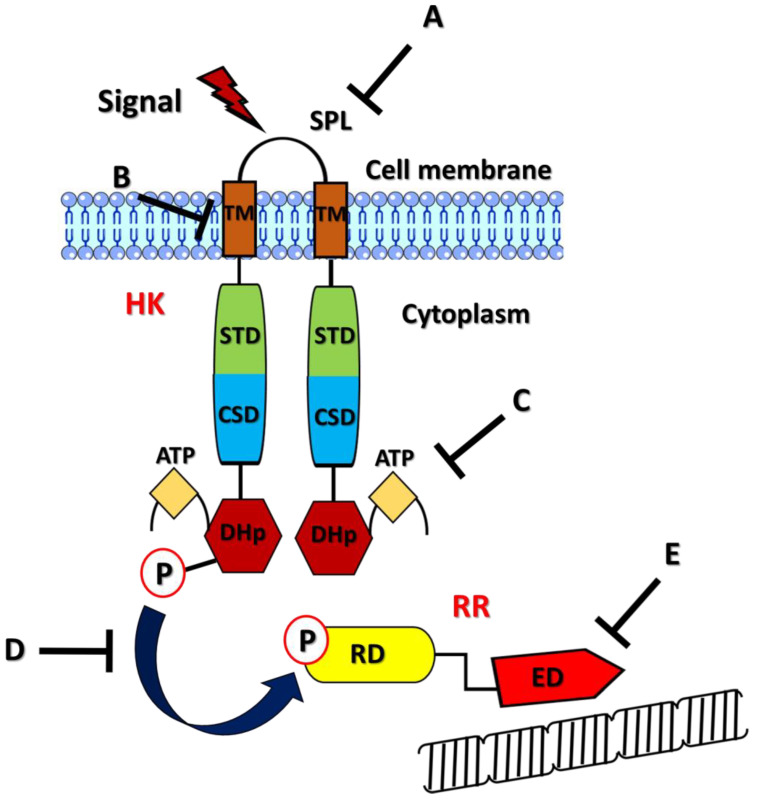
Representation of the potentially targetable components of a two-component system (TCS) for preventing MDR. The histidine kinase (HK) sensor senses a generic signal (dark red thunderbolt) via the signal peptide linker (SPL), which links two transmembrane (TM) domains. The other elements forming the HK are a signal transduction domain (STD), a cytoplasmic sensor domain (CSD), an ATP catalytic domain (ATP), and a dimerization histidine phosphotransfer domain (DHp). The response regulator (RR) is composed of the receiver domain (RD) and the effector domain (ED). The phosphorylation (P) of the HK is followed by the transfer of the phosphoryl-group (P) to the RD that induces the ED to bind to its target genes (row structure) and regulate their expression. The tipless arrows indicate the sites of action of the TCS inhibitors: (A) an inhibitor that can avoid TCS activation by its usual stimulus; (B) an inhibitor that can block HK dimerization; (C) an inhibitor targeting the site for ATP binding; (D) an inhibitor that can prevent the ATP from binding to the RD; (E) an inhibitor that can reduce or avoid the RR binding to antibiotic resistance-mediated genes.

In the last years, TCS are acquiring continuous attention because of their relevance in the various aspects of bacterial pathogenesis and antibiotic resistance [81]. The usefulness of predictive software is essential to identify, among the different species, common features, such as conserved domains of HKs and RRs, to develop new broad-spectrum antimicrobial agents, given the numerous cases of TCS-induced resistance mechanisms across some Gram-negative and Gram-positive bacteria [356,357]. The functional activities of TCS that might be targeted include (i) the inhibition of the binding properties of the sensor kinase or (ii) the response regulator, (iii) interruption of the triggering signal, and (iv) blockade of intracellular signaling or (v) HK dimerization (Figure 3) [358]. Furthermore, inhibition of the HK may be specifically enhanced based on the precise role played by TCS in physiological or pathogenic conditions since each TCS inhibitor can be used as a bacteriostatic, bactericidal, or anti-biofilm agent. An additional interesting feature is the absence of HK proteins in mammalian cells, which have other types of kinases, such as serine/threonine kinases [359]. Inhibition of the sensor kinase may be obtained, for example, by targeting the auto-phosphorylation site and/or the phosphorylation of the cognate regulator as in the case of the ATP-competitor Thienopyridine (TEP), used against some sensors in *S. pneumoniae* or other inhibitors used against several Gram-positive and Gram-negative bacteria such as *S. aureus*, *E. faecalis*, *S. mutans*, *E. coli,* and *S. enterica* [360,361,362,363,364]. Inhibitors of the phosphorylation state of a response regulator are, for example, Lactoferricin B, which has been tested against the BasR and CreB response regulators in *E. coli* [365], and an anthraquinone compound that targets the conserved phosphorylation site of the PhoP regulator [366]. 

## 6. Efflux Pumps Inhibitors: An Overview

**Figure 4:**
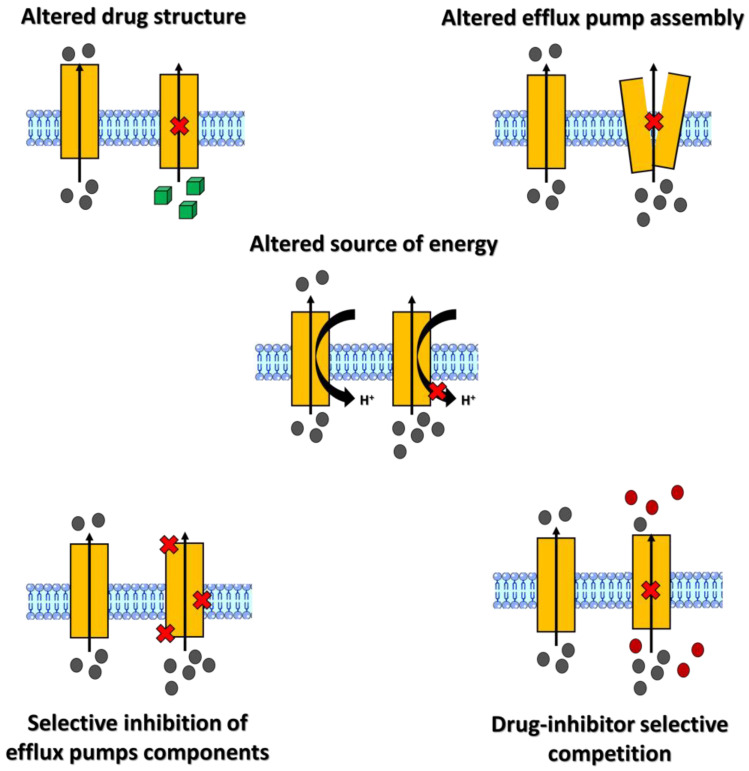
Principal mechanisms for inhibiting multidrug efflux pumps. An efflux pump is schematically represented as an orange rectangle crossing the bacterial membrane. A generic antimicrobial agent is represented as a grey dot. The flux of the pump substrate is depicted as an arrow. The red cross represents the inhibition of the substrate transfer along the pump or of a constituent of the transporter or the source of energy. (**Top left**) Intracellular accumulation of an antimicrobial drug with a conformation different (green square) from that for which the pump is able to expel; (**top right**) increased presence of the drug inside the cells due to the blockage of the efflux pump assembly; (**middle**) inhibition of the proton force mediating the efflux pump functions; (**lower left**) intracellular increase in the antimicrobial drug following the selective inhibition of some of the efflux pump components that can be in the outer membrane, the inner membrane, or the cytoplasmic side; (**lower right**) intracellular accumulation of the antimicrobial drug following the passage of a competitive inhibitor across the efflux pump channel.

Several efforts have been made and should be further improved to discover new molecules that can directly fight against antibiotic resistance-mediating efflux pumps (commonly known as efflux pump inhibitors, or EPIs). The possible inactivation of an efflux pump may be gained by (i) developing antibiotics that are not recognized as substrates, (ii) inhibiting the functional association of each component of the efflux pump, (iii) avoiding substrate binding to the active site of the pump, (iv) altering the source of energy responsible for pump activation and, lastly, (v) promoting the down-regulation of the efflux pump following the alteration of the genetic regulatory systems (Figure 4) [367]. Efflux pump inhibition should have the following features: (i) the inhibitor must not be an antibiotic, (ii) the inhibiting molecule should not target the efflux pump of the host, (iii) the inhibitor compound should comply with the safety indices of ADMET (Absorption, Distribution, Metabolism, Excretion, and Toxicity) [368]. Among the inhibitors that promote the decoupling of energy and efflux processes, the synthetic inhibitor IITR08027, for example, was reported to deactivate the resistance of *A. baumannii* overexpressing the MATE efflux pump by making the proton gradient not functional [368]. The PAβN (Phenylala-nine-Arginine-β-Naphtylamide) is, instead, a synthetic inhibitor of the RND family pumps and has been shown to be useful in strengthening the effect of levofloxacin against *P. aeruginosa* overexpressing MexAB, MexCD, and MexEF pumps by reducing their capacity to directly bind to their substrates [369]. The small molecule Verapamil, instead, is effective against *Mycobacterium tuberculosis* that up-regulates MATE pumps [370,371], while similar mechanisms of action have been seen with the molecule 1-(1-napthylmethyl)-piperazine (NMP) used together with levofloxacin against efflux pumps AcrAB and AcrEF overexpressing *E. coli* strains [372].

In in vitro studies performed with *A. baumannii* multiresistant strains showing efflux pump-mediated resistance, Riparin-B (Rip-B) was able to positively modulate norfloxacin’s effects [373]. Natural compounds have been also shown to be interesting inhibitors of the activity of efflux pumps with the aim of reducing the MIC of antimicrobial drugs [374,375]. Berberine (BBR), as a natural compound, has been, for example, proposed as an inhibitor of the MFS efflux pump MdfA in *E. coli* since it succeed in the significant increase in intracellular ciprofloxacin in resistant bacteria [376].

## 7. Conclusions and Outlook

Efflux pumps, overexpressed by resistant isolates, are continuously found and described as active ejectors of numerous antibiotics, commonly administrated in clinical practice. Efflux pump expression is under the fine genetic control of different regulatory systems; among them, TCS play a significant role. TCS are widely distributed in the microbial world and, in the future, their selective inhibition may be advantageous since they represent targets that are structurally different from those of the current antibiotics to which bacteria are not susceptible. The discovery of these new compounds and their screening have been mainly analyzed in vitro and/or in silico. For this reason, the relevance of the proposed TCS inhibitors and the new ones may be better investigated in vitro in order to carefully verify their behavior in terms of pharmacokinetic properties. Furthermore, it may be important, for example, to analyze, in antibiotic-resistant isolates with sequencing technologies, the gene encodings for HKs and RRs with the aim of creating a comprehensive antibiotic resistance database focused on TCS since the onset of point mutations in those genes may be involved in their abnormal activation associated with multidrug resistance.

## Figures and Tables

**Table 1:** Examples of antibiotic resistance mediated by TCS.

TCS	Antibiotic Resistance	Microorganism	**Refs.**
PhoQ/PhoP *	Resistance to aminoglycosides Resistance to cationic peptides Modification of lipid A	*P. aeruginosa*; *Klebsiella pneumoniae*; *Salmonella enterica*	[82,83,84,85]
**GacS/GacA**	Increased biofilm formation	*P. aeruginosa*	[86]
**PmrA/PmrB**	Resistance to cationic peptides LPS modification Modification of lipid A	*P. aeruginosa*; *K. pneumoniae*; *A. baumannii*	[87,88,89,90]
**CprS/CprR**	Resistance to cationic peptides	*P. aeruginosa*	[91,92]
**CrrA/CrrB**	Resistance to colistin	*K. pneumoniae*	[93]
**ColS/ColR**	Resistance to polymyxin	*P. aeruginosa*	[94]
**PprA/PprB**	Resistance to aminoglycosides	*P. aeruginosa*	[95,96]
**CbrA/CbrB**	Resistance to polymyxin, ciprofloxacin, and tobramycin	*P. aeruginosa*	[97]
**BfmS/BfmR**	Resistance to meropenem and colistin	*A. baumannii*	[98,99]
**CreC/CreB**	Resistance to β-lactams	*P. aeruginosa*	[100]
**CopS/CopR**	Resistance to imipenem	*P. aeruginosa*	[101]
**RcsBCS**	LPS modification	*S.enterica*	[102,103]
**GraS/GraR**	Resistance to cationic peptides	*S. aureus*	[104]
VraS/VraR *	Resistance to vancomycyn and oxacillin	*S. aureus*	[105,106]
**CesR/CesK**	Increased expression of cell envelope genes	*L. monocytogenes*	[107,108]
**ZraPR/ZraPS**	Resistance to fluoroquinolones, aminoglycosides, aminonucleosides, cyclines, and β-lactams	*E. coli*	[109]
BasR/BasS *	Resistance to class IIa bacteriocins	*E. coli*	[110]
CpxA/CpxR *	Resistance to β-lactams and aminoglycosides	*E. coli*	[111]

* TCS are involved in efflux pump-dependent antibiotic resistance and are described in this paper.

## Data Availability

Not applicable.
